# The relationship between proposed maintaining mechanisms of the transdiagnostic model of eating disorders and eating disorder symptom severity in a community sample

**DOI:** 10.1186/2050-2974-2-S1-O5

**Published:** 2014-11-24

**Authors:** Stephanie Wade, Karina Allen, Sue Byrne

**Affiliations:** 1 School of Psychology, University of Western Australia, Perth, Australia

## 

The transdiagnostic theory of eating disorders proposes that clinical perfectionism, core low self-esteem, interpersonal problems and mood intolerance can maintain eating disorder psychopathology in some individuals. The current study aimed to explore eating disorder symptom severity as a function of these maintaining mechanisms in a community sample. Participants completed questionnaires online and were classified according to whether they met criteria for zero, one, or two or more mechanisms. A greater number of maintaining mechanisms was found to be significantly associated with higher global eating disorder examination questionnaire (EDE-Q) scores, and a higher prevalence of binge eating, purging and driven exercise. Moreover, participants with 2 or more maintaining mechanisms had more severe eating disorder symptoms than participants with 1, who in turn had more severe symptoms than participants with no maintaining mechanisms. Regression models evaluated the role of each maintaining mechanism in predicting different aspects of eating disorder pathology. Self-esteem, perfectionism and mood intolerance all predicted unique variance in global EDE-Q scores. There were differences in the maintaining mechanisms that predicted binge eating, purging and driven exercise. These results provide support for the transdiagnostic model of eating disorders and have implications for detecting and treating eating disorders in community samples.

This abstract was presented in the **Peter Beumont Young Investigator award finalist** stream of the 2014 ANZAED Conference.

